# Exploring the Association Between Cigarette Consumption and Health‐Related Quality of Life: Insights From University Students in Bangladesh

**DOI:** 10.1002/puh2.70310

**Published:** 2026-07-03

**Authors:** Noushin Nohor, Md. Mahadi Hassan, Md. Fakhrul Islam Maruf, Md. Barkulla Al Bari Tain, Tanaa Mohammad Jumana, Ramisha Hoque Ikra, Sadia Akter, Yeasin Akash, Anika Bushra Boitchi

**Affiliations:** ^1^ Department of Public Health and Informatics Jahangirnagar University Dhaka Bangladesh

**Keywords:** cigarette, health‐related quality of life, smoking, university students

## Abstract

**Background:**

Cigarette smoking remains a major public health challenge and is increasingly prevalent among young adults in low‐ and middle‐income countries (LMICs). Although the long‐term health consequences of smoking are well established, its association with the health‐related quality of life (HRQoL), especially among university students, is less explored from the perspective of LMICs like Bangladesh.

**Objective:**

This study aimed to examine the association between daily cigarette consumption and HRQoL among Bangladeshi university students.

**Methods:**

A cross‐sectional study was conducted among 487 university students. HRQoL was assessed using the pre‐validated Bengali version of the SF‐36 scale, with lower scores indicating poorer quality of life. Daily cigarette consumption was categorized as nonsmoker (0), 1–3 cigarettes/day, 4 and 5 cigarettes/day, and ≥6 cigarettes/day. Unadjusted and adjusted linear regression models were used to evaluate the association between smoking intensity and HRQoL, controlling for relevant sociodemographic factors.

**Results:**

A significant association was observed between cigarette consumption and HRQoL. Compared with heavy smokers (≥6 cigarettes/day), nonsmokers had significantly higher HRQoL scores (adjusted *β* = 22.0, 95% confidence interval [CI]: 17.0–27.0, *p* < 0.001). Participants smoking 1–3 cigarettes/day (adjusted *β* = 17.0, 95% CI: 11.0–24.0, *p* < 0.001) and 4–5 cigarettes/day (adjusted *β* = 12.0, 95% CI: 4.9–18.0, *p* < 0.001) also demonstrated progressively better HRQoL. The findings indicate worsening HRQoL with increasing smoking intensity.

**Conclusion:**

Daily cigarette consumption shows a strong and graded inverse association with HRQoL among university students. These results highlight a significant association between the number of cigarettes consumed and the HRQoL among university students, underscoring the importance of early smoking reduction and cessation strategies targeting young adults.

## 1 Introduction

Tobacco use remains one of the leading causes of preventable disease and premature mortality worldwide [1]. Despite global control efforts, approximately 1.1–1.2 billion individuals continue to smoke, contributing to nearly 7 million deaths annually [1, 2]. Smoking is the third leading risk factor for disability‐adjusted life years (DALYs) and accounts for about 13.6% of global deaths [3]. In addition to active smoking, exposure to secondhand smoke poses a substantial public health burden, with more than 600,000 deaths attributed to passive exposure in 2012 alone [4].

Beyond its established role in chronic diseases, cigarette smoking adversely affects functional, psychological, and social well‐being. Smokers report reduced energy, increased fatigue, poorer sleep quality, and higher levels of anxiety and depression compared with nonsmokers [5]. These multidimensional impacts are captured by health‐related quality of life (HRQoL), a subjective measure reflecting how health status influences daily functioning, mood, and overall well‐being. HRQoL is a strong predictor of mortality and an important outcome for public health and economic evaluations [6]. Evidence consistently shows a relationship whereby heavier smoking is associated with progressively poorer HRQoL across physical and mental domains [5, 7]. Even nonsmokers exposed to environmental tobacco smoke experience lower HRQoL, particularly women exposed at home [4]. The impact of smoking on HRQoL among young adults remains relatively underexplored, especially in low‐ and middle‐income countries (LMICs) [8].

Young smokers often remain in a subclinical phase, where functional impairments are subtle and easily overlooked [9]. University students represent a critical population, as smoking prevalence is relatively high during this transitional life stage, and smoking is independently associated with anxiety, depression, and impaired HRQoL in this group [5, 6]. University students represent a particularly vulnerable population for tobacco use because the transition to university life is often accompanied by increased academic pressure, social influences, financial stress, and greater independence in health‐related decision‐making [10, 11, 12, 13]. Smoking during this period has been associated with poorer academic performance, reduced psychological well‐being, and unhealthy lifestyle behaviors that may persist into adulthood [14]. Given that health behaviors established during young adulthood frequently shape long‐term health trajectories, understanding the relationship between cigarette consumption and HRQoL among university students is important for informing targeted prevention and tobacco‐control interventions.

Bangladesh is among the 10 countries that together account for nearly two‐thirds of the world's smokers, with most users initiating daily smoking before age 25 [1, 2]. Cultural norms, gender roles, and socioeconomic factors strongly influence smoking behaviors and health perception, potentially shaping HRQoL reporting [7, 15]. Despite this burden, evidence on the association between cigarette consumption and HRQoL among Bangladeshi university students is scarce. This study aims to address this gap by examining the relationship between daily cigarette consumption and HRQoL, highlighting early subclinical impairments relevant for prevention and tobacco control strategies.

## 2 Methods

### 2.1 Study Design

The cross‐sectional study was conducted among four universities in Dhaka city in the month of May 2025. Data collection was conducted using a structured questionnaire, including socio‐demographic, smoking‐related, and HRQoL questions. To evaluate the participants’ HRQoL, we used the pre‐validated Bengali version of the SF‐36 [16]. In addition, the reliability of the SF‐36 was also ensured for the current study. In a pilot study, among the 50 participants of Jahangirnagar University, Cronbach's Alpha for the Bengali version of SF‐36 was 0.81, making the instrument reliable for use in a similar context. However, these data were not included in the final analysis. In addition, in the final analytical sample, the SF‐36 demonstrated good internal consistency (Cronbach's *α* = 0.78). The socio‐demographics data collected in the study were gathered from the previous literature conducted on a similar study population in Bangladesh [17, 18].

### 2.2 Sample Size and Technique

For the participant recruitment, a non‐probabilistic, convenience sampling technique was applied. The minimum estimated sample size for this study was calculated using the following Cochran's formula for sample size determination:

$$n=\frac{Z^{2}\times p\times \left(1-p\right)}{d^{2}}=\frac{{\left(1.96\right)}^{2}\times 0.60\times \left(1-0.60\right)}{{\left(0.05\right)}^{2}}\approx 369$$

where *Z* = 1.96 in the 95% confidence interval (CI), *p* is the prevalence of smoking in university students, and *d* is the error of margin (0.05). The prevalence (*p*) was taken as 60%, corresponding to the highest smoking prevalence reported among university students in Bangladesh by Hossain et al. [19]. The highest reported prevalence was selected to provide a conservative sample size estimate and ensure adequate precision for the overall study population. So the minimum required sample for our study was 369. However, we included a total of 487 participants in our study. As the achieved sample size exceeded the minimum required sample, the study benefited from improved statistical precision and increased power to detect significant associations.

### 2.3 Study Population

The university students studying in Bangladesh were recruited for the study. Inclusion criteria included any students who are currently enrolled in any course in a university, both public and private universities were included. However, the medical college students, despite being in a tertiary institution, were excluded from the study. Only participants who willingly participated and provided consent for participation were included in the study.

### 2.4 Data Collection

Potential participants were first approached and invited to participate in the study. After obtaining both verbal and written informed consent, trained data enumerators collected data through face‐to‐face interviews. Participants aged below 18 years were enrolled only after obtaining written informed consent from their parent or legal guardian in compliance with the ethical clearance.

### 2.5 Statistical Analysis

The HRQoL was evaluated using the overall score attained from the SF‐36 questionnaire. The SF‐36 scale has eight domains: bodily pain (BP), role physical (RP), role emotional (RE), general health (GH), mental health (MH), physical functioning (PF), social functioning (SF), and vitality (VT). The total score for each domain ranges from 0 to 100. For this study, an overall HRQoL composite score was calculated by averaging the scores of the eight SF‐36 domains. This composite score ranged from 0 to 100, with higher scores indicating better HRQoL. This measure was used as a study‐specific summary indicator and does not represent the standard SF‐36 physical component summary (PCS) or mental component summary (MCS) score. The HRQoL score is unidirectional, and with the increasing score, better quality of life is indicated for the participants. The association between smoking intensity and HRQoL was evaluated using multiple linear regression, with a *p* value of <0.05 (95% CI) considered statistically significant. In the final analysis, only the complete responses from the participants were included, removing the requirement of data imputation or handling of missing values. The adjusted multiple linear regression results were interpreted using the coefficients (*β*) obtained for each category, where positive *β* indicated a positive association between HRQoL and the particular category. The open‐sourced RStudio and R 4.4.3 were used for data analysis.

### 2.6 Operational Definition

#### 2.6.1 Smoking Status

Smoking status was classified on the basis of participants’ current smoking behavior at the time of the survey. Participants who reported currently smoking cigarettes were categorized as smokers, whereas those who reported not currently smoking were categorized as nonsmokers. The questionnaire did not distinguish former smokers from never smokers; therefore, both groups were included within the nonsmoker category.

#### 2.6.2 Daily Cigarette Consumption

Daily cigarette consumption was defined as the average number of cigarettes smoked per day by participants at the time of the survey. On the basis of self‐reported smoking frequency, participants were categorized into four groups:

- 0 cigarettes/day
- 1–3 cigarettes/day
- 4–5 cigarettes/day
- ≥6 cigarettes/day

The category of 0 cigarettes/day comprised participants who were not smoking at the time of the study, including the former smokers, whereas the remaining categories represented increasing levels of cigarette consumption among current smokers. Participants who reported occasional or non‐daily cigarette use were classified within the 1–3 cigarettes/day category if their average cigarette consumption was greater than zero but less than four cigarettes per day.

## 3 Results

Table 1 summarizes the sociodemographic and smoking characteristics of the 487 study participants. The majority of respondents were aged 18–25 years (79%), whereas 15% were older than 25 years and 6% were younger than 18 years. Regarding educational status, most participants were enrolled in their second year (32%) or third year (27%), followed by first year students (21%), fourth year students (13%), and postgraduate students (6.4%). More than half of the participants (52%) resided in university halls, 25% lived off campus, and 23% lived with their families. Nearly half of the respondents reported urban permanent residence (47%), followed by suburban (31%) and rural areas (22%). The sample was predominantly unmarried (85%), and more than half reported a monthly household income greater than BDT 21,000 (57%). Most participants were in nuclear families (72%), whereas 28% reported living in extended‐family structures. In terms of smoking‐related characteristics, 30% of participants were current smokers, whereas 70% reported being nonsmokers. Among the total sample, 11% reported smoking 1–3 cigarettes/day, 9.7% smoked 4–5 cigarettes daily, and 8.4% consumed six or more cigarettes per day. Overall, the findings indicate that the study population was largely composed of young, unmarried university students from urban backgrounds, with a substantial proportion reporting active cigarette smoking.

**TABLE 1:** Sociodemographic and smoking characteristics of the study participants (*N* = 487).

Characteristic	*N* = 487^a^
Age (years)	
<18	29 (6.0%)
>25	72 (15%)
18–25	386 (79%)
Gender	
Female	236 (48%)
Male	251 (52%)
Educational level	
First year	103 (21%)
Second year	157 (32%)
Third year	131 (27%)
Fourth year	65 (13%)
Postgraduate	31 (6.4%)
Residency	
Hall	253 (52%)
Home (outside campus)	120 (25%)
Home with family	114 (23%)
Permanent residence	
Rural	109 (22%)
Sub‐urban	150 (31%)
Urban	228 (47%)
Marital status	
Married	72 (15%)
Unmarried	415 (85%)
Monthly household income (BDT)	
<12,500	90 (18%)
12,500–21,000	120 (25%)
>21,000	277 (57%)
Family structure	
Extended	135 (28%)
Nuclear	352 (72%)
Smoking status	
Nonsmoker	343 (70%)
Smoker	144 (30%)
Daily cigarette consumption	
0	343 (70%)
1–3	56 (11%)
4–5	47 (9.7%)
≥6	41 (8.4%)


^a^ *n* (%).

**FIGURE 1:**
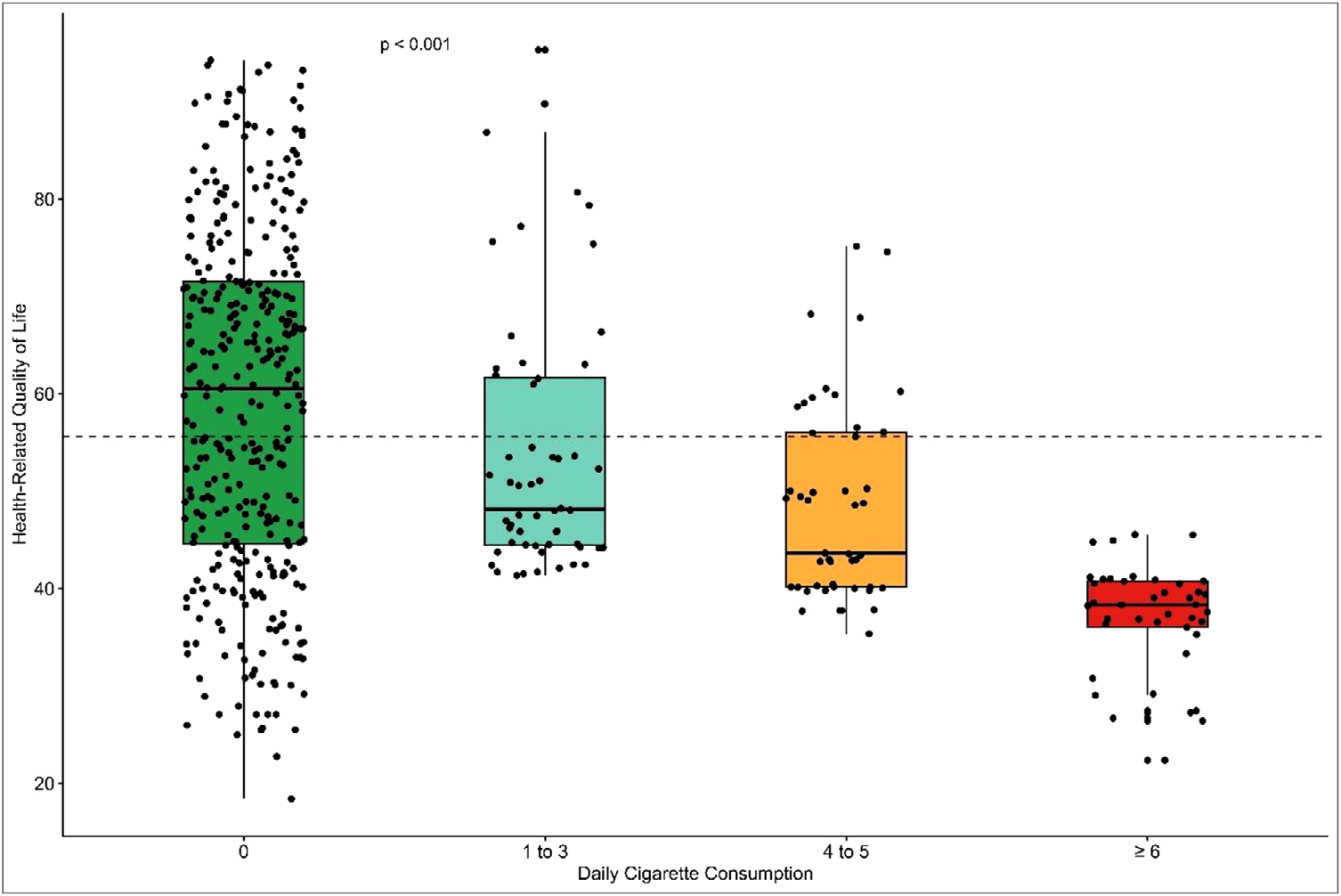
Distribution of HRQoL among different cigarette consumer groups.

Figure 1 illustrates the distribution of HRQoL across categories of daily cigarette consumption. Visual inspection suggests an inverse relationship between cigarette consumption and HRQoL, with a lower quality of life observed among participants reporting higher daily cigarette use. This pattern was statistically supported by a one‐way analysis of variance, which demonstrated significant differences in mean HRQoL across consumption groups (*F*(3), 483) = 26.69, *p* < 0.001, *η* ^2^ = 0.14), indicating a large effect size. These findings suggest that increasing levels of cigarette consumption are associated with substantial variation in HRQoL. However, to account for potential confounding factors, the final inference was based on multiple linear regression (adjusted) analyses (Table 2).

**TABLE 2:** Unadjusted and adjusted effects of daily cigarette consumption on health‐related quality of life (*N* = 487).

		Bivariate regression			Multiple regression		
Characteristic	*N*	*β*	95% CI	*p* value	*β*	95% CI	*p* value
Age (years)	487						
<18		Ref.					
>25		−0.89	−8.5, 6.7	0.8	11	4.1, 19	**0.002** ^*^
18–25		−1.4	−7.8, 4.9	0.7	11	4.7, 18	**<0.001** ^*^
Gender	487						
Female		Ref.					
Male		1.1	−1.3, 3.1	0.4	1.3	−2.1, 3.8	0.31
Educational level	487						
First year		Ref.					
Second year		6.8	2.8, 11	**<0.001** ^*^	6.2	2.0, 10	**0.004** ^*^
Third year		8.3	4.0, 13	**<0.001** ^*^	9.3	4.9, 14	**<0.001** ^*^
Fourth year		5.7	0.33, 11	**0.038** ^*^	4.4	−0.90, 9.7	0.10
Postgraduate		11	3.2, 18	**0.006** ^*^	7.2	0.40, 14	**0.038** ^*^
Residency	487						
Hall		Ref.					
Home (outside campus)		1.6	−2.0, 5.1	0.4	1.2	−2.6, 4.9	0.5
Home with family		−2.7	−6.3, 0.97	0.2	−0.43	−4.3, 3.4	0.8
Permanent residence	487						
Rural		Ref.					
Sub‐urban		−2.8	−6.7, 1.1	0.2	−0.60	−4.8, 3.6	0.8
Urban		0.13	−3.5, 3.8	>0.9	4.2	0.27, 8.1	**0.036** ^*^
Marital status	487						
Married		Ref.					
Unmarried		1.8	−2.6, 6.3	0.4	4.4	0.08, 8.7	**0.046** ^*^
Monthly household income (BDT)	487						
<12,500		Ref.					
>21,000		9.7	5.8, 14	**<0.001** ^*^	13	9.3, 17	**<0.001** ^*^
12,500–21,000		0.64	−3.7, 5.0	0.8	1.2	−3.2, 5.6	0.6
Family structure	487						
Extended		Ref.					
Nuclear		−0.67	−3.8, 2.4	0.7	0.28	−3.1, 3.7	0.9
Daily cigarette consumption	487						
≥6		Ref.					
0		19	14, 24	**<0.001** ^*^	22	17, 27	**<0.001** ^*^
1–3		17	11, 24	**<0.001** ^*^	17	11, 24	**<0.001** ^*^
4–5		9.9	3.5, 16	**0.003** ^*^	12	4.9, 18	**<0.001** ^*^

Abbreviation: CI, confidence interval.

* Significant values (*p* < 0.05) are in bold.

**FIGURE 2:**
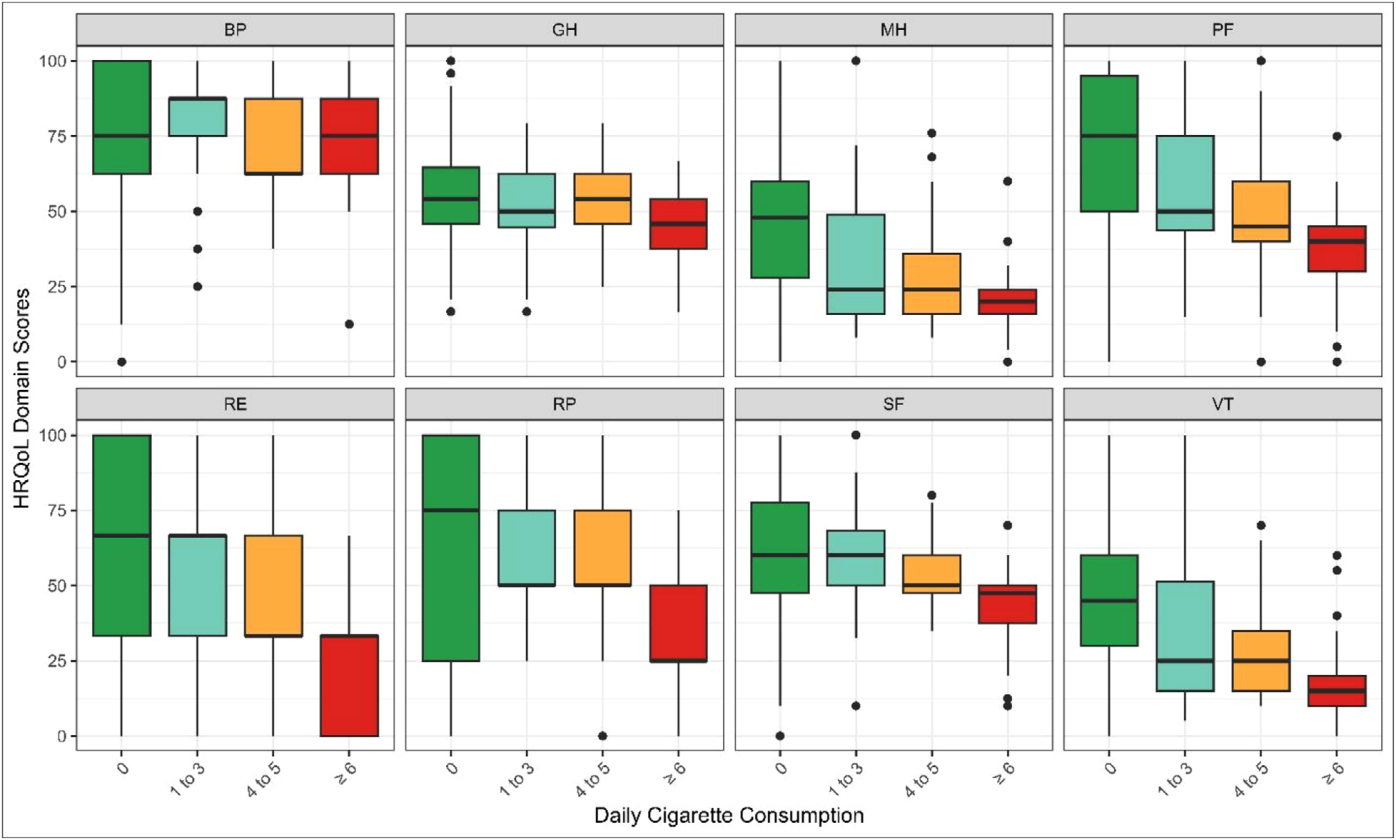
Distribution of other HRQoL domains among different cigarette consumer groups. BP, bodily pain; GH, general health; HRQoL, health‐related quality of life; MH, mental health; PF, physical functioning; RE, role emotional; RP, role physical; SF, social functioning; VT, vitality.

In addition, the distribution of other HRQoL domains across different cigarette consumer groups was also explored (Figure 2). The figure illustrates the distribution of HRQoL domain scores across different levels of daily cigarette consumption. Overall, HRQoL scores show a declining trend with increasing cigarette use. Nonsmokers generally have higher median scores across most domains, whereas participants consuming ≥6 cigarettes/day tend to report lower scores, particularly in MH, VT, PF, and SF. Similar reductions are also observed in the RE and RP domains, suggesting greater physical and emotional limitations among heavier smokers. In contrast, the BP and GH domains show comparatively smaller differences across consumption groups. Overall, the findings suggest that higher cigarette consumption is associated with poorer HRQoL.

From both simple and multiple linear regression (*R* ^2^ = 0.44, adjusted *R* ^2^ = 0.38), a significant association between daily cigarette consumption and HRQoL scores was observed (Table 2). Using heavy smokers (≥6 cigarettes/day) as the reference group, nonsmokers demonstrated substantially higher HRQoL scores in both unadjusted (*β* = 19.0, 95% CI: 14, 24, *p* < 0.001) and adjusted analyses (*β* = 22.0, 95% CI: 17, 27, *p* < 0.001). Participants who smoked 1–3 cigarettes/day also had significantly higher HRQoL scores compared with heavy smokers (unadjusted *β* = 17.0, 95% CI: 11, 24, *p* < 0.001; adjusted *β* = 17.0, 95% CI: 11, 24, *p* < 0.001). Similarly, those consuming 4–5 cigarettes daily reported moderately higher HRQoL scores than heavy smokers (unadjusted *β* = 9.9, 95% CI: 3.5, 16, *p* = 0.003; adjusted *β* = 12.0, 95% CI: 4.9, 18, *p* < 0.001). Overall, HRQoL scores decreased progressively with increasing levels of cigarette consumption, indicating a robust and significant graded association whereby higher daily cigarette intake was associated with worse HRQoL.

## 4 Discussion

Our findings demonstrate a strong and graded association between daily cigarette consumption and HRQoL, with higher smoking intensity associated with progressively lower HRQoL scores. Specifically, compared with heavy smokers (≥6 cigarettes/day), participants who were nonsmokers or smoked fewer cigarettes per day reported significantly better HRQoL scores in both unadjusted and adjusted models. This pattern suggests that even incremental reductions in daily cigarette use may be linked with an increase in perceived well‐being among young adults.

These results are consistent with existing literature showing that smoking adversely impacts quality of life in a dose‐dependent manner. A study conducted on the general population in Iran found an inverse relationship between HRQoL and smoking exposure, where heavy smokers had significantly lower HRQoL scores than the medium and light smokers [20]. Similar studies have been conducted in China [21], France [22], and Serbia [23]; all reported results align with ours. The observed direction of association aligns with well‐established evidence on the broader health effects of tobacco. Smoking intensity has been linked not only to reduced quality of life but also to significant declines in quality‐adjusted life years and increases in morbidity and mortality [24, 25]. For example, analyses of US adults showed that individuals smoking more cigarettes per day accumulate fewer quality‐adjusted life years over their lifespan compared to lighter smokers or nonsmokers, emphasizing the cumulative deleterious effects of heavier smoking [25]. Moreover, smoking is implicated in a range of chronic conditions that inevitably compromise physical, mental, and social dimensions of health, contributing to lower HRQoL [26].

The observed graded association suggests that cigarette consumption and HRQoL are closely related among university students. These findings support the importance of smoking prevention and cessation efforts in this population; however, longitudinal studies are needed to determine whether changes in cigarette consumption are associated with subsequent changes in HRQoL. This is particularly relevant in younger populations, who may underestimate the near‐term impacts of smoking on daily functioning and well‐being. Encouraging evidence also indicates that reductions in smoking intensity are associated with health benefits, including longer life expectancy and improved GH outcomes. From a public health perspective, the findings highlight an important challenge among university students, a population undergoing a critical developmental transition where health behaviors are often established and maintained into adulthood. The observed association between higher cigarette consumption and poorer HRQoL suggests that the burden of smoking extends beyond future disease risk and may affect students’ day‐to‐day well‐being. Given the substantial prevalence of smoking among young adults in Bangladesh, universities represent a strategic setting for tobacco‐control interventions. Strengthening smoke‐free campus policies, expanding access to smoking cessation support, and implementing targeted health promotion programs may help reduce tobacco use and support the overall well‐being of university students.

## 5 Strengths and Limitations

The key strength of our study lies in demonstrating a clear relationship between HRQoL and multiple categories of cigarette use. However, the cross‐sectional design limits causal inference, and self‐reported smoking intensity may be subject to reporting or recall bias. The adjustment of the multiple linear regression was also less comprehensive in our study. In addition, formal diagnostic assessments of multiple linear regression assumptions, including residual normality, homoscedasticity, influential observations, and multicollinearity, were not conducted. Therefore, the robustness of the estimated associations should be interpreted with caution. As the HRQoL score included MH along with other dimensions, the relevant non‐sociodemographic factors, such as anxiety, depression, sleep, and broader psychosocial functioning, were not controlled in the multiple regression. In addition, future longitudinal studies in similar populations would strengthen the understanding of the temporal relationships between smoking reduction and improvements in HRQoL.

## 6 Conclusion

This study demonstrates a clear and significant association between daily cigarette consumption and HRQoL, with heavier smoking associated with progressively worse HRQoL. A graded pattern was observed whereby participants reporting lower cigarette consumption generally demonstrated higher HRQoL scores than heavier smokers, highlighting that the adverse effects of smoking extend beyond clinical outcomes to everyday well‐being. These findings underscore the importance of smoking reduction and cessation strategies, particularly among young adults. The findings from our study support the importance of smoking prevention and cessation strategies among young adults. However, longitudinal studies are required to determine whether reductions in cigarette consumption can lead to improvements in HRQoL over time.

## Author Contributions

**Noushin Nohor**: conceptualization, methodology, writing – original draft, writing – review and editing. **Md. Mahadi Hassan**: formal analysis, data curation, writing – original draft, writing – review and editing. **Md. Fakhrul Islam Maruf**: writing – review and editing. **Md. Barkulla Al Bari Tain**: writing – review and editing. **Tanaa Mohammad Jumana**: writing – review and editing. **Ramisha Hoque Ikra**: writing – review and editing. **Sadia Akter**: writing – review and editing. **Yeasin Akash**: writing – review and editing. **Anika Bushra Boitchi**: writing – review and editing.

## Funding

The authors have nothing to report.

## Ethics Statement

This study was conducted in accordance with the Helsinki Declaration. The study received ethical approval from the “Biosafety, Biosecurity, and Ethical Committee of Jahangirnagar University (Ref No: BBEC, JU/M 2025/04 (225))” before data collection.

## Consent

All participants in this study provided written informed consent for the publication of their data in anonymized form. They were assured that no identifying personal information would be published. Participants were informed of their right to withdraw consent at any time, without affecting their involvement in the study.

## Conflicts of Interest

The authors declare no conflicts of interest.

## Transparency Statement

The lead author, Noushin Nohor, affirms that this manuscript is an honest, accurate, and transparent account of the study being reported; that no important aspects of the study have been omitted; and that any discrepancies from the study as planned (and, if relevant, registered) have been explained.

## Data Availability

The datasets used and analyzed during the current study are available from the corresponding author upon reasonable request.
